# Beyond self‐report: The relationship between daily social media use and sleep in university students

**DOI:** 10.1111/bjhp.70093

**Published:** 2026-07-13

**Authors:** Hayley Breare, Thomas McAlpine, Jessica Charlesworth, Patrick Clarke, Barbara A. Mullan

**Affiliations:** ^1^ School of Population Health Curtin University Bentley Western Australia Australia; ^2^ Behavioural Science and Health Research Group Curtin University Bentley Western Australia Australia; ^3^ EnAble Institute Curtin University Bentley Western Australia Australia

**Keywords:** objective measures, repeated measures, sleep, social media, students

## Abstract

**Objectives:**

University students regularly report insufficient sleep, with almost one‐third of students achieving less than 6.5 hours per night. Social media is one behaviour shown to negatively influence sleep. However, there has been a reliance on self‐report measures of both processes, which may not reflect objective behaviour. The aim was to determine if there is a relationship between social media use and sleep outcomes when measured objectively, and if so, which sleep outcomes were associated with social media use.

**Design:**

A longitudinal repeated measures design was used to assess daily social media use and sleep outcomes over 14 days.

**Methods:**

Participants wore accelerometers to measure their sleep, with total daily social media use derived from smartphone data.

**Results:**

Sixty‐two participants (*M*_age_ = 22.11 years, SD = 5.82 years) completed the study. Linear mixed models revealed no between or within‐participant effects of daily social media on sleep duration, sleep onset latency, sleep efficiency and bedtime. However, TikTok showed a positive between‐participants effect on bedtime. A generalized linear mixed model also revealed that Snapchat had a negative between‐participants effect on sleep onset latency.

**Conclusions:**

Findings indicate that the duration of time spent on social media does not impact sleep across this sample. However, interactions with different social media platforms such as TikTok and Snapchat may play an important role in influencing sleep quality. These findings suggest that behavioural measures do not reflect the same pattern of effects observed with self‐report, highlighting the need for future research to corroborate findings derived from self‐report.

**Statement of Contribution**

**What is already known on this subject?**

- It is well‐recognized that university students experience poor sleep.
- Social media use may impact students' sleep quality.
- However, much of the literature focuses on self‐report methods.

**What does this study add?**

- Social media use was not related to sleep outcomes when objectively measured.
- Individual social media platforms, however, may uniquely impact sleep.
- Self‐report methods pose concerns regarding the robustness of past findings.

## INTRODUCTION

The prevalence of sleep difficulties among adults is estimated to affect 20%–45% of the adult population (Chattu et al., 2018; Hillman & Lack, 2013). Quality sleep is critical to sustaining physical health and mental well‐being (Chattu et al., 2018). Unsurprisingly, when people consistently experience poor sleep, it can contribute to negative health outcomes such as chronic health conditions (e.g., diabetes, obesity, heart disease), poor psychological well‐being (e.g., anxiety, depression) and impaired cognitive performance and decision making (Chattu et al., 2018). Insufficient sleep has been highlighted as a public health concern, which often goes unrecognized, with broad implications to health, well‐being and economic consequences (Hafner et al., 2017; Lim et al., 2023).

University students are commonly recognized for being an at‐risk population for sleep difficulties, due to their need to balance increased academic, social and employment demands (Patrick et al., 2017). University students consistently report regular sleep disturbances, including shorter total sleep time, delayed sleep onset latency (i.e., the time it takes to fall asleep), later wake times and experience overall low sleep quality (Becker et al., 2018; Lund et al., 2010). Poor sleep among students may be particularly detrimental to learning and academic performance and can increase their susceptibility to poor physical and mental health (Gao et al., 2019; Gomes et al., 2011; Patrick et al., 2017). As university students are among the heaviest users of social media, this may play a specific role in the sleep patterns commonly observed within this population (Kolhar et al., 2021).

There are more than 5 billion users of social media worldwide (Digital & Meltwater, 2024). In January 2024, globally, adults spent an average of 6 h 40 min per day on the internet, with an average of 2 h 23 min spent on social media platforms (Digital & Meltwater, 2024). Social media fulfils the need for social engagement and is highly accessible, particularly among young people (Kim et al., 2016). Social media platforms comprise algorithms that are used to capture attention and encourage prolonged user engagement (Zhang et al., 2019). The need to feel connected alongside the urgency to respond to incoming messages (Vorderer et al., 2016) may keep individuals at a heightened state of alertness throughout the evening and bedtime period. As a result, this ongoing engagement with social media may lead to further difficulties falling asleep or staying asleep at nighttime.

Few studies to date have used objective measures such as wrist‐worn accelerometers when looking at the association between social media and sleep among young adults (Das‐Friebel et al., 2020; McAlpine et al., 2023), raising concerns about the reliance on self‐report (Althubaiti, 2016; Schoeller, 1995). Studies that have used self‐reports have found a negative relationship between social media and sleep (Alonzo et al., 2021; Brautsch et al., 2023). However, there is not always an agreement between subjective and objective measures, indicating that individuals may tend to either under‐ or overestimate their behaviours (Allom et al., 2016; Todd et al., 2022). Mahalingham et al. (2023) found very weak, non‐significant associations between subjective and objective measures of social media use. These findings are consistent across studies and raise concerns regarding the validity of self‐report methodologies (Parry et al., 2021). This highlights a need to corroborate the relationship between social media and sleep using objective measures.

Among research examining the impact of social media on sleep, few studies have sought to examine the influence of individual social media platforms. This may be important given the observation that, across various platforms, there are differences between active and passive use of social media on well‐being (Masciantonio et al., 2021). Interestingly, Azhari et al. (2022) found that those with poorer sleep quality engaged more in active posting on Facebook; however, this type of association was not found for other social media platforms such as Instagram, Snapchat and Twitter. Similarly, Garett et al. (2018) found that frequent posting on Twitter was associated with poor sleep quality, specifically during the week. The use of different social media platforms varies based on individual characteristics, where emotional, cognitive, social and habitual factors may influence daily fluctuations in social media use (Wang et al., 2012). Hence, it is important that longitudinal designs are used to capture these variations, particularly in social media use.

Many studies that have examined the relationship between social media and sleep outcomes have relied on cross‐sectional designs (Alonzo et al., 2021). However, the effects of social media behaviours may depend on individual differences, where true effects may be obscured when examining overall population effects. Longitudinal designs permit the examination of both within‐ (i.e., whether individuals' fluctuations in social media use correspond with day‐to‐day variations in sleep) and between‐participant effects (i.e., whether individuals who use more social media on average have poorer sleep on average) (Curran & Bauer, 2011). Previous research has demonstrated the utility of within‐subject designs, offering greater precision in tracking changes over time than can be shown in only examining between‐subject effects (Armstrong‐Carter et al., 2023). Without separating these effects, it is unclear whether there is a true relationship between social media and sleep quality. Therefore, the aim of the present study was to use a longitudinal design to identify if there is a relationship between social media use and sleep outcomes when measured objectively. It was hypothesized that there would be both a between‐participant and within‐participant effect of social media use (i.e., overall and across individual platforms) on sleep outcomes across 14 days. Secondly, we also aimed to identify which sleep outcomes were associated with overall social media use and use of individual platforms, both between and within participants.

## MATERIALS AND METHODS

### Participants and procedure

A longitudinal repeated measures design was used to examine both between‐ and within‐participant effects of social media use on sleep quality indicators across a 14‐day period. Ethics approval was obtained from the University's Human Research Ethics Committee (HRE2020‐0696). Data was collected over 4 months between August and October 2021. A total of 62 undergraduate students were recruited through a student participant pool from an Australian university. Participants were required to be over the age of 18 years and use at least one of the five chosen social media platforms (Facebook, Instagram, Twitter, TikTok, Snapchat) to participate in the study. At the completion of the study, participants were awarded course credit.

Informed consent was obtained from participants prior to completing baseline measures. One week prior to the study, participants were instructed to ensure they were able to record their daily social media use before being able to commence the study. For Android users, participants were instructed to download *‘App Usage’* (V5.20 or above), a mobile application that is used to show individual application usage. For Apple users, participants were instructed to ensure ‘recording of application usage’ was turned on in the built‐in screen time mobile feature. Participants attended an initial induction session where they completed baseline surveys and were provided with their wrist‐worn accelerometer, to be worn for the next 14 days to measure their sleep activity. They were also shown step‐by‐step instructions on how to access and provide their social media use via screenshots of their data. The baseline measure consisted of demographics and questions related to sleep hygiene behaviours, which were part of a larger study (McAlpine et al., 2023). Participants were also asked to provide screenshots of their last 7 days of phone and social media use. Over the following 2 weeks, participants wore their accelerometer each night and were sent a daily SMS at their identified wake time. This SMS contained a link to their personalized sleep diary, which asked questions about the previous night's sleep. Participants were contacted at the end of weeks 1 and 2 to provide their screenshots of social media usage, which were sent via email to the researcher.

### Measures

#### Demographics

Age, gender, ethnicity, level of education, whether participants had any children aged 2 years or younger living in their household, any previously diagnosed sleep disorder and whether any other devices were used for social media were measured in the baseline survey.

#### Social media use

Social media use included time spent on the following platforms: Facebook, Instagram, Twitter, TikTok and Snapchat. These social media platforms reflect the top five most popular social media platforms for 2021. We chose to exclude platforms that involved long‐form video content, blogs, forums and messaging‐only platforms (Kemp, 2021), as these platforms primarily facilitate active engagement behaviours (e.g., direct communication with others), rather than passive consumption of information (Roberts & David, 2022; Verduyn et al., 2015). Social media use was measured objectively via screenshots provided by participants of their daily social media data. This data was recorded by either *‘App Usage’* or the built‐in feature of their phone and was extracted for each day. Daily social media across all social media platforms was summed to create a total daily social media score for each day and for each platform.

#### Sleep quality

Indicators of sleep quality included sleep duration (i.e., total sleep time), sleep efficiency (i.e., time asleep compared to the proportion of time in bed), sleep onset latency and bedtime (i.e., time participants fell asleep). Indicators of sleep quality were measured objectively using ActiGraph GT9X Link © Activity Monitors (ActiGraph LLC., Pensacola, FL, USA). These activity monitors were worn as wrist watches (on the non‐dominant hand) to record activity and movement during participants' sleep each night over the 2 weeks. Each device was set at a 60‐s epoch length and a sample rate of 30 Hz, and data were collected using three axes. Data were downloaded into ActiLife Software (V6.13.4), where the Tudor‐Locke algorithm identified sleep periods (min non‐zero epoch = 15). As recommended, the Cole‐Kripke algorithm was applied to the data to analyse each participant's sleep periods (Cole et al., 1992). Actigraph devices have demonstrated high validity against other objective measures of sleep, including gold‐standard overnight polysomnography (Marino et al., 2013).

#### Consensus sleep diary

The 10‐item self‐report Consensus Sleep Diary (Carney et al., 2012) was included in the daily survey sent to participants via SMS. For example, responses to *‘What time did you get out of bed?’* were used to validate the conclusion of participants' sleep period. Example items included in the Sleep Consensus Diary include: ‘In total, how long did you sleep?’ and ‘How would you rate the quality of your sleep?’ Response formats consisted of 5‐point Likert scales ranging from ‘very poor’ (1) to ‘very good’ (5). Items related to time, required responses with the hour and minute. Consensus sleep diaries have been shown to demonstrate good validity and utility with self‐report measures of insomnia and Actigraph monitors (Maich et al., 2018). The purpose of this measure was to validate accelerometer data (Carney et al., 2012; Maich et al., 2018). This was done in line with previous research (Lee et al., 2018; Quante et al., 2018), for example, sleep periods identified through actigraphy were inspected and, where necessary, adjusted to align with self‐reported sleep data (e.g., where there were sharp changes in activity). In addition, where multiple sleep periods were detected by the algorithm, sleep periods were combined into one.

### Statistical analyses

Twelve participants were excluded due to either non‐compliance with the study protocol, device errors (i.e., phone or Actigraphy), or excessive missing data (>50%). Normality was assessed using Shapiro–Wilk tests and visual inspection of histograms and Q–Q plots. Descriptive statistics indicated substantial positive skew and kurtosis across all individual social media variables on raw variables, reflecting heavy‐tailed distributions typical of behavioural data. Shapiro–Wilk tests confirmed non‐normality for each individual platform and the composite social media use score (*p* < .001), each with a positive skew. The composite score (sum of all platforms) showed skewness (.89) and kurtosis (.59), within recommended thresholds (Tabachnick & Fidell, 2013). Although distributions of predictor variables were positively skewed, linear mixed models are robust to departures from normality with large samples and repeated measures (Schielzeth et al., 2020); therefore, no transformations were made. Extreme outliers were winsorized by bringing the value to three standard deviations (SDs) above or below the mean (Reifman & Keyton, 2010). Descriptive statistics were reported as means or medians, standard deviations or interquartile ranges (IQRs). Analyses were conducted in RStudio, using the lme4 and lmerTest packages (Bates et al., 2015; Pinheiro & Bates, 2023). Linear mixed models were employed to examine the degree to which indicators of sleep quality varied in conjunction with social media use over 2 weeks. We included each indicator of sleep quality as the dependent variable in four separate models. For each model, demographic variables were first entered to test their association with the dependent variable. If significant, they were retained in the full model as control variables. Social media use was disaggregated into between and within‐participant effects before being added into the full model (Curran & Bauer, 2011). For each model, the participant (Participant ID) was used as the grouping variable to account for the non‐independence of measurements.

Additional analyses were conducted for each individual platform to identify any effects that may be masked by overall social media use. However, Twitter was excluded due to the extremely short average amount of time that participants spent on this platform. A separate generalized linear mixed model was conducted for sleep onset latency to account for the non‐normal distribution of outcome scores. Given that the data were exponentially distributed, the gamma family with log link function was selected to analyse models with sleep onset latency. Finally, to assess whether the association between social media and sleep outcomes differed depending on the individual, the final model for each dependent variable was compared to a random slopes model (where the effect of social media was allowed to vary by participant).

## RESULTS

### Participants demographics

Sixty‐two participants (*women = 45, men = 16, non‐binary = 1*), between 18 and 45 years of age (*M* = 22.11 years, SD = 5.82 years), completed the study. The highest level of education of most participants was high school (69.4%). Most participants self‐identified as Caucasian (62.9%), Asian (29%), or other (6.5%). Two participants had a child under the age of two living at home, and four participants (6.5%) identified as being diagnosed with a sleep disorder (i.e., insomnia, chronic fatigue). Sixty‐nine per cent of the sample reported exclusively using their mobile phone for social media. Across the sample, 6 participants did not use Facebook (9.68%), 5 did not use Instagram (8.06%), 25 did not use TikTok (40.32%), 43 did not use Twitter (69.35%), and 16 did not use Snapchat (25.81%). The means and SDs for users only are shown in Data S1. The daily means, standard deviations, medians and IQRs for social media and sleep are reported in Table 1. Paired samples *t*‐tests were conducted to examine any differences in social media use from baseline to the 14‐day study period, including only participants with complete data at both time points. Facebook use significantly decreased in average daily use from baseline (*M* = 20.14, SD = 24.91) to the 14‐day period (*M* = 15.90, SD = 18.25), *t*(56) = 2.27, *p* < .05, *d* = .30. No significant differences were found for other platforms (*p* > .05). Overall, there were 826 days of valid data collected out of a possible 868 days (95.2%), indicating a high level of data completeness across the 14‐day observation period.

**TABLE 1:** Descriptive statistics for key variables for daily social media use at baseline and during the 14‐day period.

Platform	Time period	*N*	Days with zero use	*M*	SD	Md	IQR
Facebook*	Baseline	391	22.3%	20.31	32.23	8.40	24.5
	14‐day	861	9.7%	17.75	25.88	8.00	22.8
Instagram	Baseline	393	12.21%	49.82	58.52	36.00	55.0
	14‐day	861	9.7%	45.04	55.56	27.60	51.0
Twitter	Baseline	423	85.8%	1.82	6.67	.00	.0
	14‐day	868	77.4%	1.51	6.41	.00	.0
TikTok	Baseline	416	57.5%	33.36	59.0	.00	49.1
	14‐day	868	50.0%	35.92	64.99	.00	51.4
Snapchat	Baseline	416	31.5%	23.31	38.17	5.00	31.2
	14‐day	861	27.4%	23.99	41.37	5.00	33.6
Total social media	Baseline	394	—	133.60	105.80	105.80	129.2
	14‐day	861	—	122.80	89.63	111.29	124.4
Screen use	Baseline	394	—	333.90	178.96	295.50	213.2
	14‐day	861	—	340.56	188.89	302.00	201.2

Sleep variables (14‐day nightly average)	*M*	SD
Sleep duration	6.57 h	1.32 h
Sleep efficiency	80.33%	8.38%
Sleep onset latency	8 mins	11 mins
Bedtime	11:58 pm	107 mins

*Note*: *N* represents the number of observations. *p <.05.

Abbreviations: IQR, interquartile range; SD, standard deviation.

### Sleep duration

The initial model was conducted with sleep duration and demographic variables. This demonstrated that gender had a significant relationship with sleep duration (*B* = 38.18, SE = 11.21, *t =* 3.41, *p* < .01). This suggests that when holding the other demographic variables constant, women slept 38.18 min longer than men on average. When predictor variables were added to the model, neither the between‐ nor within‐participant social media use was associated with sleep duration (see Table 2). Further examination of individual social media platforms also demonstrated a non‐significant result for all social media platforms for between and within‐participant effects, as shown in Table 3. Comparison of these models to random slopes models showed no significant differences (*χ* ^2^ = .13, df = 2, *p* = .93). Thus, there was no evidence that either total social media use or the use of individual platforms was associated with total sleep duration either within participants or across the sample.

**TABLE 2:** Linear mixed model of sleep duration (in minutes) as predicted by between and within social media use.

		Variance	SD	ICC
Random effects				
Participant (intercept)		1149	33.90	.19

	*B*	SE	*t*	*p*
Fixed effects (between)				
Intercept*	363.00	9.70	37.41	<.001
Gender*	39.67	10.98	3.62	<.001
Total social media use	−3.77	5.14	−.07	.47
Fixed effects (within)				
Total of social media	2.39	2.64	.90	.37

Abbreviatins: ICC, intraclass correlations; SD, standard deviation.

* *p* < .05.

**TABLE 3:** Linear mixed model of sleep duration (in minutes) as predicted by between and within‐individual social media platforms.

		Variance	SD	ICC
Random effects				
Participant (intercept)		1218.00	34.90	.20

	*B*	SE	*t*	*p*
Fixed effects (between)				
Intercept*	363.88	10.09	36.08	<.001
Gender*	38.65	11.48	3.37	<.001
Facebook	.07	.28	.25	.81
Instagram	−.04	.12	−.33	.75
TikTok	−.06	.10	−.56	.58
Snapchat	−.04	.16	−.25	.80
Fixed effects (within)				
Facebook	.07	.17	.41	.68
Instagram	.02	.09	.23	.82
TikTok	.07	.08	.82	.41
Snapchat	−.08	.10	−.81	.42

Abbreviations: ICC, intraclass correlations; SD, standard deviation; SE, standard error.

* *p* < .05.

### Sleep efficiency

The initial model included sleep efficiency and demographic variables, revealing no significant effect of demographics on sleep efficiency. Therefore, demographic variables were excluded in subsequent analyses. When predictor variables were included in the model, there were no significant effect of between (*t* = .04, *p* = .96) or within social media use (*t* = 1.60, *p* = .11) on sleep efficiency. This indicated that there is a consistent null effect of social media use and sleep efficiency across participants, regardless of the day. This null finding also did not seem to depend on the individual when social media use was included as a random slope (*χ* ^2^ = .12, df = 2, *p* = .94). To ensure that effects were not masked by overall usage, individual social media platforms for between‐ and within‐participant effects of Facebook, Instagram, TikTok and Snapchat were included in a separate model. This also revealed no significant effects on sleep efficiency (all *p* > .05). Both models are shown in Tables 4 and 5.

**TABLE 4:** Linear mixed model of sleep efficiency as predicted by between and within social media use (standardized).

		Variance	SD	ICC
Random effects				
Participant (intercept)		37.55	6.13	.53

	*B*	SE	*t*	*p*
Fixed effects (between)				
Intercept*	80.26	.81	99.69	<.001
Total social media use	.03	.81	.04	.97
Fixed effects (within)				
Total of social media	.35	.22	1.60	.11

Abbreviations: ICC, intraclass correlations; SD, standard deviation; SE, standard error.

* *p* < .05. Total social media use was standardized to aid model convergence.

**TABLE 5:** Linear mixed model of sleep efficiency as predicted by between and within individual social media platforms (in minutes).

		Variance	SD	ICC
Random effects				
Participant (intercept)		37.82	6.15	.54

	*B*	SE	*t*	*p*
Fixed effects (between)				
Intercept*	80.27	.81	99.32	<.001
Facebook	.01	.04	.30	.76
Instagram	.01	.02	.73	.47
TikTok	−.02	.02	−1.13	.26
Snapchat	.03	.02	1.03	.31
Fixed effects (within)				
Facebook	.001	.01	.11	.91
Instagram	.005	.01	.66	.51
TikTok	.01	.01	1.31	.19
Snapchat	.01	.01	.87	.38

Abbreviations: ICC, intraclass correlations; SD, standard deviation; SE, standard error.

* *p* < .05.

### Sleep onset latency

An initial generalized linear mixed model conducted with sleep onset latency as the dependent variable and demographics revealed having a sleep disorder was significantly related to the time it took participants to fall asleep (*B* = −.39, SE = .15, *t =* −2.44, *p* < .05). This suggests that participants who did not have a sleep disorder on average took 32% less time to fall asleep. Including sleep disorder as a control variable accounted for participants with difficulties with sleep onset and other sleep‐related issues. When between‐ and within‐variables of social media use were added to the model, there was no main between‐participant (*t* = −.67, *p* = .50) or within‐participant effect (*t* = −1.62, *p* = .11) on sleep onset latency, as shown in Table 6. However, when individual social media platforms were included in the model as predictors (see Table 7), there was a significant between‐participant effect of Snapchat use on sleep onset latency (*B* = −.19, SE = .07, *t* = −2.80, *p* < .05). This indicated that greater average use of Snapchat between individuals was associated with decreased sleep onset latency by 18%, suggesting overall individuals with more use will on average fall asleep quicker than those with less use.

**TABLE 6:** Generalized linear mixed model of sleep onset latency as predicted by between and within social media use (in hours).

		Variance	SD	ICC
Random effects				
Participant (intercept)		.02	.12	.01

	*B*	SE	*t*	*p*
Fixed effects (between)				
Intercept*	2.76	.32	8.73	<.001
Sleep disorder*	−.37	.16	−2.31	<.05
Total social media use	−.02	.03	−.67	.50
Fixed effects (within)				
Total social media use	−.08	.05	−1.62	.12

Abbreviations: ICC, intraclass correlations; SD, standard deviation; SE, standard error.

* *p* < .05. Sleep disorder was coded as 1 = yes, 2 = no.

**TABLE 7:** Generalized linear mixed model of sleep onset latency as predicted by between and within individual social media platforms (in hours).

		Variance	SD	ICC
Random effects				
Participant (intercept)		.004	.06	.004

	*B*	SE	*t*	*p*
Fixed effects (between)				
Intercept*	2.82	.31	9.21	<.001
Sleep disorder*	−.41	.16	−2.59	<.001
Facebook	−.10	.12	−.80	.42
Instagram	−.06	.05	−1.13	.26
TikTok	.06	.04	1.26	.21
Snapchat*	−.19	.07	−2.80	<.05
Fixed effects (within)				
Facebook	.03	.16	.22	.82
Instagram	−.05	.08	−.60	.55
TikTok	−.11	.07	−1.59	.11
Snapchat	−.06	.09	−.71	.48

Abbreviations: ICC, intraclass correlations; SD, standard deviation; SE, standard error.

* *p* < .05.

### Bedtime

Bedtime was measured as the time participants went to sleep, as indicated by the Actigraphy. The initial model included demographic variables and revealed that age (*B* = −5.92, SE = 1.76, *t = −*3.37, *p* < .01) was significantly related to the time participants went to bed. Participants who went to bed later tended to be younger than those who went to bed earlier in the night. This further indicates that for every year older the participant was, bedtime occurred on average 5.92 min earlier. When predictor variables were added to the model, there was no significant effect of between‐participant social media use on bedtime (see Table 8). However, when the individual platforms were tested separately, there was a significant between‐participant effect of TikTok on bedtime, *B* = .44, SE = 11.63, *t = 2.27, p* < .05. This reflected that overall, for participants who used TikTok 1 h more (per day) on average, their bedtimes were on average 26 min later. The full model is shown in Table 9.

**TABLE 8:** Linear mixed model of bedtime (as minutes from midnight) as predicted by between and within social media use (in minutes).

		Variance	SD	ICC
Random effects				
Participant (intercept)		826.99	75.58	.54

	*B*	SE	*t*	*p*
Fixed effects (between)				
Intercept*	826.99	42.49	19.46	<.001
Age*	−5.09	1.87	−2.72	<.05
Total social media use	.19	.15	1.29	.20
Fixed effects (within)				
Total of social media	−.05	.05	−.93	.35

Abbreviations: ICC, intraclass correlations; SD, standard deviation; SE, standard error.

* *p* < .05. Bedtime values were converted to numeric values for analysis.

**TABLE 9:** Linear mixed model of bedtime (as minutes from midnight) as predicted by between and within individual social media platforms (in minutes).

		Variance	SD	ICC
Random effects				
Participant (intercept)		838.61	44.95	.53

	*B*	SE	*t*	*p*
Fixed effects (between)				
Intercept	838.61	44.95	18.66	<.001
Age*	−5.64	1.99	−2.84	<.05
Facebook	−.04	.53	−.07	.94
Instagram	.24	.22	1.08	.28
TikTok*	.44	.19	2.27	<.05
Snapchat	−.49	.32	−1.57	.12
Fixed effects (within)				
Facebook	−.01	.17	−.07	.94
Instagram	−.06	.09	−.63	.53
TikTok	−.06	.08	−.76	.45
Snapchat	.12	.10	1.20	.23

Abbreviations: ICC, intraclass correlations; SD, standard deviation; SE, standard error.

* *p* < .05. Bedtime values were converted to numeric values for analysis.

### Sensitivity analysis

A sensitivity analysis was conducted using weekday‐only data to determine whether the associations (and absence of associations) between social media use and sleep outcomes were consistent when weekend data were excluded. Overall, the pattern of results was broadly similar to the primary analyses (see Data S2). However, within‐person TikTok use was significantly associated with sleep duration *B* = .19, SE = .09, *t* = 2.08 and *p* < .05, and the association between Snapchat and sleep onset latency was no longer statistically significant, *B* = −.02, SE = .09, *t* = −.23 and *p* = .82. This suggests that individuals who used TikTok longer on any given weekday, slept slightly longer (i.e., 11 s longer sleep duration for every extra minute spent on TikTok above their average use).

## DISCUSSION

The aim of the current study was to identify whether there is a relationship between social media use and sleep outcomes when measured objectively. Our findings demonstrated that when looking at daily social media use, there was no significant between‐ or within‐participant association with any of the four sleep outcomes examined. However, when looking at individual social media platforms, Snapchat had a between‐participant effect on sleep onset latency, and TikTok had a between‐participant effect on bedtime. These between‐participant effects indicate that individuals with higher average Snapchat use fell asleep faster, while those with higher average TikTok use had later bedtimes. While our findings suggest that overall, combined social media use does not influence sleep outcomes, they do highlight that individual social media platforms may share some association with specific aspects of sleep.

The findings of the study are inconsistent with previous literature that has relied on self‐report methods, with previous results indicating that there is a relationship between social media use and sleep outcomes (Alonzo et al., 2021; Levenson et al., 2017; Scott et al., 2019). However, the few studies that have used objective measures have also shown similar findings to this study, demonstrating an absence of an association between social media use and sleep outcomes among students (Das‐Friebel et al., 2020). Other research, such as in screen use, has used objective camera footage to measure screen time in the 2 h before bed and demonstrated that not all types of screen time were associated with impaired sleep (Brosnan et al., 2024). Objective measures are valuable in their ability to assess true behaviours, particularly those like social media and sleep that vary considerably (Knutson et al., 2007).

Our findings, therefore, converge with other research that has begun to question the robustness of the association between social media use and other well‐being indicators (Sewall et al., 2022). As with sleep, a considerable body of research has highlighted links between social media use and psychological distress, such as depression and anxiety (Keles et al., 2020). However, consistent with the current findings, these associations are either small or non‐existent when objective measures of social media are employed (Rozgonjuk et al., 2020; Seabrook et al., 2016; Sewall et al., 2022). Together, this highlights the possibility that associations between social media and sleep outcomes may be a product of associations between an individual's perceptions or concerns about their social media use (self‐report) and not with actual use (objective measures). Although in the current study, subjective sleep quality was assessed via the CSD, research shows that the CSD can lead to overestimations of some sleep indicators compared to objective measures of sleep (Dietch & Taylor, 2021). While we did not make comparisons between these subjective and objective measures of sleep, future research should consider incorporating both measures of sleep quality indicators to develop a more comprehensive understanding of sleep quality.

The lack of association found between social media and sleep could be a result of the distinctive characteristics of university students. Our findings reflect those of Das‐Friebel et al. (2020), where the findings may highlight the distinct experiences and flexibility of university students' schedules and bedtimes, which differ from those of the general population. Variations in student schedules may provide them with more flexibility around their sleep period, depending on their class start times (Onyper et al., 2012). Research on social media and sleep has typically focused on adolescents who have more restrictions around sleep and wake time (Alonzo et al., 2021). In our sample, participants on average went to bed late (i.e., around midnight) and were only marginally below the recommended 85% sleep efficiency benchmark (Reed & Sacco, 2016), suggesting perhaps they had a greater opportunity to adapt to their university schedules, regardless of their time spent on social media.

Interestingly, the flexibility in university students' sleep schedules may increase their susceptibility to bedtime delays following prolonged social media use. Our findings revealed that participants who had greater average TikTok use per day tended to have, on average, later bedtimes. This is not unsurprising given that TikTok's short‐form videos, combined with their targeted algorithms, have been observed to elicit prolonged user engagement (Zhang et al., 2019). One explanation for our finding is that if TikTok was used prior to sleep, it may have displaced part of the sleep period, resulting in later bedtimes. TikTok has been shown to be difficult to disengage from, leading to high levels of inattention and potential bedtime procrastination (Hill et al., 2024; Maulida et al., 2024). Previous research has also shown that students often attribute bedtime delays to social media use (Kolhar et al., 2021). Our findings highlight the importance of the cognitive mechanisms that occur during the use of TikTok, which may be particularly important when approaching the sleep period. Further, the intentions behind social media use may also play an important role in sleep outcomes. For example, TikTok has been shown to be used to combat boredom at bedtime, which has been shown to be a predictor of bedtime procrastination (Hill et al., 2024; Teoh et al., 2021). While we did not measure the timing of TikTok use, future research should consider understanding the cognitive mechanisms that exist around TikTok use during the 30‐min to 2‐h period prior to sleep.

Lastly, we found that participants who, on average, spent more time on Snapchat showed, on average, less time to fall asleep. In contrast, this is inconsistent with other research that has shown self‐reported Snapchat use to be associated with poorer sleep outcomes (Bergfeld & Van den Bulck, 2021). While this result is not clear, this association could be due to several reasons. For example, compared to other social media, Snapchat is more focused on communication with family and friends (Vaterlaus et al., 2016). Perhaps Snapchat interactions with others evoke positive or negative valence, which may play into the sleep period. Rzewnicki et al. (2020) found that positive experiences of social media use were not associated with sleep disturbances, which may contribute to shorter sleep onset latency. Alternatively, participants in the current study only used Snapchat for a short period of time throughout the day. However, we cannot be sure if this finding is a result of Snapchat use itself or this type of interactive behaviour with such applications (e.g., frequent ‘checking’) that may confound this relationship.

A key limitation of the study is that social media use was assessed as total daily usage, without specific information related to the timing of social media use. Timing of social media has been shown to be an important factor, with previous research indicating relationships with sleep outcomes (Brautsch et al., 2023). For example, Massar et al. (2021) demonstrated that heavier smartphone use before bed (3 h prior) was associated with later sleep timings with objective measures. Social media use during the sleep period may demonstrate different mechanisms, such as sleep displacement, bright light exposure and hyperarousal, influencing sleep outcomes (Cain & Gradisar, 2010). This limits the generalizability of the current findings to contexts where social media use occurs at night or in proximity to sleep. Future research should include measures of temporal patterns of social media use to determine whether associations with sleep outcomes differ between daytime and nighttime use. In addition, a further limitation of the study was the inability to make comparisons between weekend and weekday social media use and sleep patterns. Nonetheless, sensitivity analyses showed a similar pattern of findings across sleep outcomes for weekdays alone; however, they revealed a within‐person effect for TikTok and sleep duration, and a non‐significant effect of Snapchat on sleep onset latency. These differences may have contributed to variability in the observed associations across analyses, particularly given that university students often show different sleep patterns on weekends compared to weekdays (Vestergaard et al., 2024). These findings suggest that students may compensate for extended weekday TikTok use by sleeping longer the following day. Future studies with longer data collection periods that allow for more balanced weekday and weekend comparisons could examine these differences more systematically, to determine whether associations between social media use and sleep outcomes are moderated by day type (e.g., weekday vs weekend).

Lastly, results for the individual platforms should be interpreted with caution, as there were a substantial number of observations where some participants had zero social media use for specific platforms, which contributed to the skewed distributions. These scores, however, do reflect real patterns of social media engagement. The observed distribution suggested that social media use is not uniformly distributed across days, pointing to important social media use patterns that warrant future investigation. Future research could consider examining binge patterns, characterized by intensive engagement on specific days with subsequent minimal or no use patterns, to capture the fluctuating nature of social media engagement and its potential differential effects on sleep.

## CONCLUSION

The current study demonstrated that when measured objectively, social media use showed no association with sleep outcomes. However, our findings indicate that interactions with different platforms, such as TikTok and Snapchat, may influence sleep in different ways. Future research is needed to understand the timing of social media use in relation to various sleep outcomes. To the best of our knowledge, this study is the first to use objective measures of both social media use and indicators of sleep quality to examine the relationship between social media use and sleep among students. Using objective measures to assess day‐to‐day temporal associations emphasizes the need to move beyond self‐report methods to disaggregate between and within effects. The distinction between these levels of analysis is essential, as between‐person effects identify individual differences in susceptibility based on typical usage patterns, while within‐person effects capture whether day‐to‐day fluctuations actually precipitate changes in sleep (Curran & Bauer, 2011). Our null within‐person findings suggest that daily variations in social media use may not directly impact sleep quality in the immediate timeframe, though stable individual differences in platform engagement patterns may still be relevant. The current study provides insight into the growing body of literature suggesting that behavioural measures of social media do not reflect the same pattern of effects observed with self‐report measures and therefore emphasizes the importance of incorporating such measures in future research to corroborate the findings derived from self‐report.

## AUTHOR CONTRIBUTIONS

**Hayley Breare:** Conceptualization; methodology; data curation; formal analysis; investigation; visualization; writing – original draft; writing – review and editing; project administration. **Thomas McAlpine:** Conceptualization; methodology; data curation; formal analysis; investigation; visualization; writing – review and editing; supervision; funding acquisition; project administration. **Jessica Charlesworth:** Writing – review and editing; writing – original draft. **Patrick Clarke:** Conceptualization; methodology; writing – review and editing; supervision. **Barbara A. Mullan:** Conceptualization; methodology; writing – review and editing; supervision.

## Supporting information


Data S1.

Data S2.

## Data Availability

The data supporting this article cannot be made publicly available due to the conditions of our ethics approval. Participants were advised that access to their information would be limited to the research team and, if required for audit or investigation, staff from the Curtin University Office of Research and Development. As such, we are unable to make data publicly available.
