# Supplementary material for: Beyond self‐report: The relationship between daily social media use and sleep in university students
Source: Br J Health Psychol. 2026 Jul 13;31(3):e70093. doi: 10.1111/bjhp.70093 (PMC13358886; doi:10.1111/bjhp.70093)
Supplement: Supplementary file 1 — Data S1. [file BJHP-31-0-s002.docx]

**Supplementary Materials 1**

***Overall daily means and standard deviations for all participants compared to only those who used the platform***

|  |  | ***All users*** | | | | **Only those who used the platform** | | |
| --- | --- | --- | --- | --- | --- | --- | --- | --- |
| **Platform** | **Social media use** | **N** | ***M*** | ***SD*** | ***Within SD*** | **N** | ***M*** | ***SD*** |
| **Facebook** | 14-Day | 62 | 17.75 | 25.88 | 17.68 | 62 | 19.67 | 26.54 |
| **Instagram** | 14-Day | 62 | 45.04 | 55.56 | 22.09 | 57 | 49.91 | 56.04 |
| **Twitter** | 14-Day | 62 | 1.51 | 6.41 | 1.50 | 7 | 6.70 | 12.16 |
| **TikTok** | 14-Day | 62 | 35.92 | 64.99 | 20.22 | 37 | 71.85 | 76.63 |
| **Snapchat** | 14-Day | 62 | 23.99 | 41.37 | 11.36 | 46 | 33.16 | 45.42 |
| **Total social media use** | 14-Day | 62 | 122.80 | 89.63 | 43.31 | 62 | - | - |

*Note. N represents total number of participants included in the sample.*
