# Supplementary material for: Beyond self‐report: The relationship between daily social media use and sleep in university students
Source: Br J Health Psychol. 2026 Jul 13;31(3):e70093. doi: 10.1111/bjhp.70093 (PMC13358886; doi:10.1111/bjhp.70093)
Supplement: Supplementary file 2 — Data S2. [file BJHP-31-0-s001.docx]

**Supplementary Material 2**

**Linear mixed models and Generalised Linear Mixed models for weekday data**

**Sleep duration**

| *Table 1. Linear mixed model of sleep duration (in minutes) as predicted by between and within social media use on weekday only data* | | | | |
| --- | --- | --- | --- | --- |
|  |  | ***Variance*** | ***SD*** | ***ICC*** |
| ***Random effects*** |  |  |  |  |
| Participant (intercept) |  | 1307.6 | 36.16 | .22 |
|  | ***B*** | ***SE*** | ***t*** | ***p*** |
| ***Fixed effects (between)*** |  |  |  |  |
| Intercept* | 359.95 | 10.58 | 34.02 | < .001 |
| Gender* | 38.34 | 11.92 | 3.22 | <.01 |
| Total social media use | −5.62 | 4.57 | −1.23 | .22 |
| ***Fixed effects (within)*** |  |  |  |  |
| Total of social media | 3.59 | 4.20 | .85 | .40 |
|  |  |  |  |  |
| *Note. ICC = Intraclass correlations, * p < .05*   \| *Table 2. Linear mixed model of sleep duration (in minutes) as predicted by between and within individual social media platforms on weekday only data* \| \| \| \| \| \| --- \| --- \| --- \| --- \| --- \| \|  \|  \| ***Variance*** \| ***SD*** \| ***ICC*** \| \| ***Random effects*** \|  \|  \|  \|  \| \| Participant (intercept) \|  \| 1333 \| 36.51 \| .22 \| \|  \| ***B*** \| ***SE*** \| ***t*** \| ***p*** \| \| ***Fixed effects (between)*** \|  \|  \|  \|  \| \| Intercept* \| 360.53 \| 10.87 \| 33.17 \| <.001 \| \| Gender* \| 37.98 \| 12.38 \| 3.07 \| <.001 \| \| Facebook \| .06 \| .30 \| .20 \| .84 \| \| Instagram \| -.00 \| .12 \| -.01 \| .99 \| \| TikTok \| -.18 \| .12 \| -.66 \| .10 \| \| Snapchat \| -.01 \| .17 \| -.09 \| .93 \| \| ***Fixed effects (within)*** \|  \|  \|  \|  \| \| Facebook \| -.01 \| .21 \| -.48 \| .63 \| \| Instagram \| -.07 \| .10 \| -.74 \| .46 \| \| TikTok* \| .19 \| .09 \| 2.08 \| <.05 \| \| Snapchat \| -.04 \| .11 \| -.30 \| .76 \| \| *Note. ICC = Intraclass correlations, * p < .05* \| \| \| \| \| | | | | |

| *Table 3. Linear mixed model of sleep efficiency as predicted by between and within social media use (standardised) on weekday data* | | | | |
| --- | --- | --- | --- | --- |
|  |  | ***Variance*** | ***SD*** | ***ICC*** |
| ***Random effects*** |  |  |  |  |
| Participant (intercept) |  | 37.61 | 6.13 | .57 |
|  | ***B*** | ***SE*** | ***t*** | ***p*** |
| ***Fixed effects (between)*** |  |  |  |  |
| Intercept* | 80.63 | .87 | 92.18 | <.001 |
| Total social media use | .29 | .79 | .37 | .71 |
| ***Fixed effects (within)*** |  |  |  |  |
| Total of social media | .30 | .33 | .93 | .35 |
|  |  |  |  |  |
| *Note. ICC = Intraclass correlations, * p < .05. Total social media use was standardised to aid model convergence.* | | | | |

**Sleep Efficiency**

| *Table 5. Linear mixed model of sleep efficiency as predicted by between and within individual social media platforms (in minutes) on weekday data* | | | | |
| --- | --- | --- | --- | --- |
|  |  | ***Variance*** | ***SD*** | ***ICC*** |
| ***Random effects*** |  |  |  |  |
| Participant (intercept) |  | 38.47 | 6.20 | .57 |
|  | ***B*** | ***SE*** | ***t*** | ***p*** |
| ***Fixed effects (between)*** |  |  |  |  |
| Intercept* | 80.64 | .89 | 90.78 | <.001 |
| Facebook | .67 | 2.67 | .25 | .80 |
| Instagram | 1.29 | 1.50 | .87 | .39 |
| TikTok | -.80 | 1.16 | -.69 | .49 |
| Snapchat | 1.57 | 1.71 | .92 | .36 |
| ***Fixed effects (within)*** |  |  |  |  |
| Facebook | -.29 | 1.04 | -.28 | .78 |
| Instagram | .49 | .63 | .78 | .44 |
| TikTok | .49 | .48 | 1.01 | .31 |
| Snapchat | -.74 | .82 | -.90 | .37 |
| *Note. ICC = Intraclass correlations, * p < .05* | | | | |

| *Table 5. Generalised linear mixed model of sleep onset latency as predicted by between and within social media use (in hours) for weekday only* | | | | |
| --- | --- | --- | --- | --- |
|  |  | ***Variance*** | ***SD*** | ***ICC*** |
| ***Random effects*** |  |  |  |  |
| Participant (intercept) |  | .05 | .23 | .06 |
|  | ***B*** | ***SE*** | ***t*** | ***p*** |
| ***Fixed effects (between)*** |  |  |  |  |
| Intercept* | 2.85 | .39 | 7.24 | <.001 |
| Sleep disorder* | -.43 | .20 | -2.21 | <.05 |
| Total social media use | -.03 | .04 | -.79 | .43 |
| ***Fixed effects (within)*** |  |  |  |  |
| Total social media use | -.04 | .05 | -.76 | .45 |
|  |  |  |  |  |
| *Note. ICC = Intraclass correlations, * p < .05. Sleep disorder was coded as 1 = yes, 2 = no.* | | | | |

**Sleep onset latency**

| *Table 6. Generalised linear mixed model of sleep onset latency as predicted by between and within individual social media platforms (in hours) for weekday only* | | | | |
| --- | --- | --- | --- | --- |
|  |  | ***Variance*** | ***SD*** | ***ICC*** |
| ***Random effects*** |  |  |  |  |
| Participant (intercept) |  | .04 | .21 | .06 |
|  | ***B*** | ***SE*** | ***t*** | ***p*** |
| ***Fixed effects (between)*** |  |  |  |  |
| Intercept* | 2.87 | .39 | 7.30 | <.001 |
| Sleep disorder* | -.43 | .20 | -2.15 | <.05 |
| Facebook | -.02 | .15 | -.12 | .91 |
| Instagram | -.08 | .07 | -1.20 | .23 |
| TikTok | .05 | .06 | .81 | .42 |
| Snapchat | -.16 | .87 | -1.82 | .07 |
| ***Fixed effects (within)*** |  |  |  |  |
| Facebook | .04 | .17 | .24 | .80 |
| Instagram | -.06 | .08 | -.76 | .45 |
| TikTok | -.07 | .07 | -1.00 | .32 |
| Snapchat | -.02 | .09 | -.23 | .82 |
| *Note. ICC = Intraclass correlations, * p < .05* | | | | |

| *Table 7. Linear mixed model of bedtime (as minutes from midnight) as predicted by between and within social media use (in minutes)* | | | | |
| --- | --- | --- | --- | --- |
|  |  | ***Variance*** | ***SD*** | ***ICC*** |
| ***Random effects*** |  |  |  |  |
| Participant (intercept) |  | 6470 | 80.44 | .60 |
|  | ***B*** | ***SE*** | ***T*** | ***p*** |
| ***Fixed effects (between)*** |  |  |  |  |
| Intercept* | 813.46 | 45.27 | 17.97 | <.001 |
| Age* | -4.61 | 2.00 | -2.31 | <.05 |
| Total social media use | 13.41 | 9.50 | 1.41 | .16 |
| ***Fixed effects (within)*** |  |  |  |  |
| Total of social media | -.07 | 3.51 | -.02 | .98 |
|  |  |  |  |  |
| *Note. ICC = Intraclass correlations, * p < .05. Bedtime values were converted to numeric values for analysis.* | | | | |

**Bedtime**

| *Table 8. Linear mixed model of bedtime (as minutes from midnight) as predicted by between and within individual social media platforms (in minutes)* | | | | |
| --- | --- | --- | --- | --- |
|  |  | ***Variance*** | ***SD*** | ***ICC*** |
| ***Random effects*** |  |  |  |  |
| Participant (intercept) |  | 6057 | 77.83 | .60 |
|  | ***B*** | ***SE*** | ***t*** | ***p*** |
| ***Fixed effects (between)*** |  |  |  |  |
| Intercept | 825.78 | 47.65 | 17.33 | <.001 |
| Age* | -5.19 | 2.11 | -2.46 | <.05 |
| Facebook | -.03 | .56 | -.05 | .96 |
| Instagram | .21 | .23 | .90 | .37 |
| TikTok* | .53 | .21 | 2.56 | <.05 |
| Snapchat | -.51 | .33 | -1.51 | .14 |
| ***Fixed effects (within)*** |  |  |  |  |
| Facebook | .13 | .20 | .65 | .52 |
| Instagram | .07 | .10 | .73 | .47 |
| TikTok | -.11 | .09 | -1.31 | .19 |
| Snapchat | .09 | .11 | .78 | .44 |
| *Note. ICC = Intraclass correlations, * p < .05. Bedtime values were converted to numeric values for analysis.* | | | | |
